# Towards Self-Organized Anodization of Aluminum in Malic Acid Solutions—New Aspects of Anodization in the Organic Acid

**DOI:** 10.3390/ma13173899

**Published:** 2020-09-03

**Authors:** Lidia Zajączkowska, Dariusz Siemiaszko, Małgorzata Norek

**Affiliations:** Institute of Materials Science and Engineering, Faculty of Advanced Technologies and Chemistry, Military University of Technology, Str. gen. Sylwestra Kaliskiego 2, 00-908 Warsaw, Poland; lidia.zajaczkowska@student.wat.edu.pl (L.Z.); dariusz.siemiaszko@wat.edu.pl (D.S.)

**Keywords:** aluminum, anodization, malic acid, electrolyte aging, self-ordering regime

## Abstract

In this work, aluminum (Al) anodization in malic acid electrolytes of different concentrations (0.15 M, 0.25 M, and 0.5 M) was studied. The close-packed hexagonal pore structure was obtained for the first time in this organic acid in a 0.5 M solution, at 250 V and temperature of 5 °C. Moreover, the process was investigated as a function of the number of cycles carried out in the same electrolyte. A repetition of anodization under seemingly the same external electrochemical parameters (applied voltage, temperature, etc.) induced serious changes in the electrolyte. The changes were reflected in the current density vs. time curves and were most evident in the higher concentrated electrolytes. This phenomenon was tentatively explained by a massive incorporation of malate anions into anodic alumina (AAO) framework. The impoverishment of the electrolyte of the malate anions changed internal electrochemical conditions making easier the attraction of the anions to the Al anode and thus the AAO formation. The electrolyte modification was advantageous in terms of pore organization: In a 0.25 M solution, already after the second anodization, the pore arrangement transformed from irregular towards regular, hexagonal close-packed structure. To the best of our knowledge, this is the first observation of this kind.

## 1. Introduction

Aluminium (Al) and its alloys are frequently used for lightweight structures such as aircrafts, automobiles, aerospace components and interiors, where good mechanical properties, corrosion resistance and economic considerations are of high importance. A naturally occurring thin layer of passive oxide on Al surface makes it resistant to corrosive environment. To increase the inherent corrosion resistance of Al, the metal (and its alloys) is subjected to anodization [1,2,3]. After the electrochemical process, a thick and porous anodic alumina is formed on Al surface with a characteristic barrier layer that separated the metal from the corrosive environment.

Apart from its excellent anti-corrosive properties, anodic aluminium oxide (AAO) is frequently used as a template for fabrication of various functional nanostructures applied in sensors, capacitors, optical devices, high density magnetic recording media, etc. [4,5,6,7]. The attractiveness of the AAO template for nanofabrication results from the ability to precisely control its thickness, pore diameter (*D_p_*) or interpore distance (*D_c_*) by simple changing electrochemical parameters, such as applied voltage, temperature, anodizing time, or the electrode distance [8,9]. While *D_p_* can be additionally regulated (increase) by post-processing pore broadening in acidic solutions, the *D_c_* is fixed when the anodization is completed and is mainly determined by the anodization voltage. For a given electrolyte, there is always a threshold voltage above which a stable anodization cannot be continued, owing to the appearance of burning phenomena [10,11]. This fact, in turn, limits the size of possible *D_c_* in AAO. Furthermore, in order to obtain a regular pore arrangement with close-packed hexagonal structure, the anodization has to be carried out under so-called self-organized conditions. The self-organizing conditions are operative within a narrow processing window pertinent for a given electrolyte. So far, highly ordered AAO with *D_c_* in the range of 80–500 nm was produced during mild anodization in sulfuric, oxalic, phosphoric or etidronic acid [12,13,14,15]. Regular AAO with *D_c_* up to ≈400 nm was also produced during hard anodization, which involves high current/high voltage conditions preceded by pre-anodization under mild anodization [16,17]. Yet, production AAO with *D_c_* > 500 nm remains still very challenging since the self-organizing conditions need to be operative under high-voltage anodization (U > 250 V) where the process breakdown (dielectric breakdown) can easily occur.

It was shown before that anodization in organic acid solutions with low dissociation constants can open the new route for fabrication of highly ordered AAO with large pore intervals [18]. Ono et al. [19] studied anodization in organic acid electrolytes and demonstrated that high-current-density anodization is required for a regular pore growth. Moreover, it was shown that low electrolyte temperature and relatively high electrolyte concentration are important for maintaining self-ordering growth of AAO. The self-organized AAO was realized in malonic acid at 120V and tartaric acid at 195 V with *D_c_* of 300 and 500 nm, respectively. Recently, the self-ordered alumina with large period (up to ca. 900 nm) was produced during anodization in citric acid solutions [20,21]. The anodization was conducted in high citric acid solution (1.5 M), low temperature (0 °C) and high voltage (400 V). It was noticed that the process of pore nucleation differs substantially from previously observed phenomena and was associated with a very slow pore nucleation. Furthermore, a massive incorporation of citric anions into the alumina framework under the self-ordering conditions was recorded, which was manifested by the change of the AAO colour from grey to black.

Malic acid has similar molecular structure to that of citric acid, however, it has smaller molecular mass and larger dissociation constant (*K_a_* = 9.1 × 10^−3^) as compared to the latter one (*K_a_* = 7.45 × 10^−4^) [22]. Therefore, it can be expected that the self-organizing anodization will be realized under lower voltage, since the larger the *K_a_*, the smaller the critical voltage that guarantees a stable anodization without the burning phenomena [23]. So far, there are only two reports on the aluminium anodization in malic acid [24,25]. Chu et al. [25] was able to conduct a stable anodization at 450 V, 283 K, and 2 wt.%. Kikuchi et al. [24] studied Al anodization at 250 V, 25 °C and 0.5 M and demonstrated that in this process the pore growth initiates at the grain boundaries. Only after prolonged anodization time, depending on the applied voltage, the growth region extended to the entire aluminium surface. However, highly ordered AAO could not be obtained under the anodization conditions applied in those works. Therefore, more studies are needed to find and understand the anodization in the malic acid solutions.

Here, we present a systematic study on the anodization of Al in three different concentrations of malic acid, at different temperatures and applied voltages up to 300 V. The obtained AAO templates were green-to-violet, suggesting their complex chemical composition. It was shown that, in agreement with previous findings, the lower the temperature and the higher the concentration of organic acid, the closer are the conditions to self-ordering regime. The close-packed hexagonal pore structure with *D_c_* ≈ 530 nm was formed in 0.5 M solution, at 250 V and temperature of 5 °C. Moreover, the anodization in the organic acid was investigated as a function of cycles in the same electrolyte. It was demonstrated that, most probably due to a considerable incorporation of Al containing malate complexes into the AAO framework, the electrolyte is changing (aging) very fast. As a consequence, in relatively high concentrated malic acid solutions (0.25 M, 0.5 M), the current vs. time transients were not reproduced on each and subsequent anodizing process performed under the same external electrochemical parameters (applied voltage, temperature, concentration of electrolyte, etc.). On the other hand, the electrolyte modification appeared to be advantageous in terms of pore organization: In a 0.25 M solution already after the second anodization cycle, the pore arrangement transformed from circular and irregular structure towards regular, hexagonal close-packed arrays. The results presented in this work can be important also for anodization carried out in other organic acid electrolytes.

## 2. Materials and Methods

High-purity Al foil (99.9995% Al, Goodfellow, UK) with a thickness of about 0.25 mm was cut into rectangular specimens (2 cm × 1 cm). Before the anodization process, the Al foils were degreased in acetone and ethanol and subsequently electropolished in a 1:4 mixture of 60% HClO_4_ and ethanol at 0 °C, under constant voltage of 25 V, for 2.5 min. Next, the samples were rinsed with a distilled water, ethanol and dried. As-prepared Al specimens were insulated at the back and the edges with acid-resistant tape to avoid burning, and served as the anode. A Pt grid was used as a cathode and the distance between both electrodes was kept constant (ca. 5 cm). Pt/Al electrode area ratio was about 25. DL-malic acid 99+%, was purchased from Acros Organics (Morris Plains, NJ, USA). A large, 1 L electrochemical cell and cooling bath thermostat (model MPC-K6, Huber company, Offenburg, Germany) were employed in the anodizing process. An adjustable DC power supply with voltage range of 0–300 V and current range of 0–5 A, purchased from NDN, model GEN750_1500 TDK Lambda, TDK Co. Tokyo, Japan, was used to control the applied voltage. RIGOL DM 3058E digital multimeter (RIGOL, Beaverton, OR, USA) was used to measure and transfer the registered current to a computer. Alumina was chemically removed using a mixture of 6 wt.% phosphoric acid and 1.8 wt.% chromic acid at 60 °C for 120 min.

Morphological analysis and microanalysis of chemical composition were made using a field-emission scanning electron microscope FE-SEM (AMETEK, Inc., Montvale, NJ, USA) equipped with energy dispersive X-ray spectrometer (EDS). The chemical composition analysis was performed at 20 kV, magnification of 500, spot 1.5, and with a constant distance of samples to the detector (WD = 10). Each measurement was repeated at least three times in different places of a given sample and an average of all measurements was taken to determine the chemical composition of a studied sample. To obtain the interpore distance (*D_c_*) of the fabricated samples, Fast Fourier transforms (FFTs) were generated based on three SEM images taken at the same magnification for every sample, and were further used in calculations with WSxM software (version 5.0) [26]. The average *D_c_* was estimated as an inverse of the FFT’s radial average abscissa from three FE-SEM images for each sample.

Conductivity of the electrolytes was measured in a thermostatic cell with Elmetron CC 505 conductivity meter, Zabrze, Poland. At the end, average values from three measurements were given.

## 3. Results and Discussion

**Figure 1:**
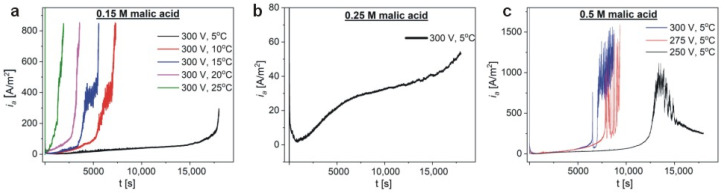
Current density (*i_a_*) versus time (t) transients for the samples anodized in 0.15 M (**a**), 0.25 M (**b**), and 0.5 M (**c**) malic acid solution.

High-purity aluminum was anodized in three malic acid concentrations: 0.15 M, 0.25 M, and 0.5 M. The Al samples were initially anodized for 5 h to assess optimal electrochemical conditions for every malic acid concentration. In Figure 1a, the current density (*i_a_*) vs. time (*t*) curves for the samples anodized in 0.15 M malic acid at 300 V and temperature (T) ranging between 5–25 °C, are shown. As can be seen, in the temperature range 10–25 °C, after a certain anodization time, the current starts to grow rapidly and almost vertically up. The onset of the *i_a_* growth is delayed with decreasing the anodization temperature. Only at 5 °C, it was possible to carry out a full 5-h-long process, therefore, this temperature was selected for the next anodization processes. In 0.25 M solution, the process was stable at 300 V and 5 °C (Figure 1b): At the initial stage a current overshoot is observed, but then the *i_a_* drops to a minimum value, and increases gradually to about 60 A/m^2^, passing through a plateau. However, no sharp current increases were observed. Next, the Al was anodized in 0.5 M malic acid solution (Figure 1c). As can be seen, under 300 and 275 V the current starts to be unstable after 1.5–2 h of anodization, which is manifested by an appearance of multiple sharp peaks, reaching the maximum at ca. 1500 A/m^2^. Therefore, the anodizing voltage was lowered to 250 V. A full, 5-h anodization recorded under 250 V is presented in Figure 1c. For the first 3 h, the *i_a_*(*t*) is relatively low (not exceeding 50 A/m^2^), but after ≈10,000 s of anodization, *i_a_*(*t*) starts to increase steeply to a value of ca. 1000 A/m^2^. After reaching the maximum, the *i_a_* decreases and tends to stabilize at a certain value.

**Figure 2:**
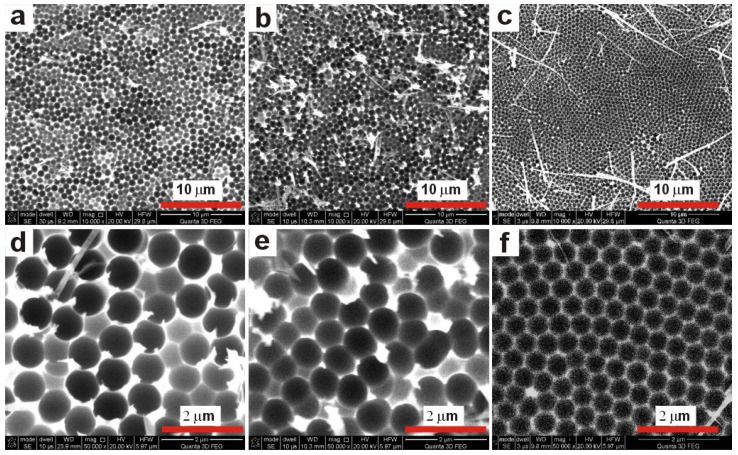
SEM images (**upper row**—magnification 10,000×, **lower row**—magnification 50,000×) of the nano-concaves left on aluminum surface after AAO dissolution obtained by a 5-h anodization at 5 °C in 0.15 M (**a**,**d**), 0.25 M (**b**,**e**), and 0.5 M (**c**,**f**) malic acid solution at 300 V (0.15 M and 0.25 M) and 250 V (0.5 M).

In Figure 2, SEM images of the Al substrates obtained after dissolution of anodic alumina (only the samples anodized for full 5 h) are shown. In the selected conditions, a self-ordering regime was achieved for the sample anodized in 0.5 M malic acid solution, at 250 V and temperature of 5 °C. In Figure 2c,f, a quite large area of close-packed hexagonal pore array (self-assembly in a hexagonal pattern) can be seen. Moreover, long aluminum nanopillars in the irregular cell junctions are also visible, which were previously observed and ascribed to the field-assisted local dissolution of barrier oxide [19,27]. The best hexagonal pore arrangement in the highest electrolyte concentration is not surprising. The highest order of two-dimensional array of holes was also observed previously during anodization in 0.5 M oxalic acid solution [28]. In contrast to the hexagonal cells in the sample anodized in 0.5 M solution, the pores formed on Al surface during anodization in 0.15 M and 0.25 M under 300 V were much more irregular and circular. The distance between the centers of dimples that correspond to the interpore distance (*D_c_*) in AAO was 758 ± 101, 665 ± 98, and 527 ± 12 nm for the samples anodized under 300 V in 0.15 M, 300 V in 0.25 M, and 250 V in 0.5 M solution, respectively.

The shape of the *i_a_*(*t*) curve for the Al specimen anodized in 0.5 M at 250 V is very similar to the one recorded by Kikuchi et al. [23]. However, the maximum *i_a_* obtained in this work was one order of magnitude greater than the one in the work of Kikuchi et al. [24] (1000 A/m^2^ as compared to 250 A/m^2^). This is quite surprising especially taking into account that the anodization temperature was much lower in this work (5 °C as compared to 20 °C in the Kikuchi work). Moreover, in the previous work, the peak of *i_a_* occurred after 5 h of anodization, whereas in this work it occurred after ca. 3 h and 40 min. What is most important though, is that previously no self-organized alumina was formed. These discrepancies may be due to a larger ratio of cathodic and anodic surface area applied in this work. When the cathode/anode ratio is relatively large, a high current can be generated [29,30]. High current/high electric field strength anodization, in turn, favours the formation of regular pore arrays [19,31,32]. Zheng et al. [30], for instance, observed the increase of current density from 3000 to 4000 A m^−2^ when the area ratio of anode to cathode increased from 1:10 to 1:50, although the concentration of phosphoric acid was decreased [30]. In this work, the Pt/Al surface area ratio was ca. 25 and high current density generated under those conditions apparently forced pores to assembly into close-packed hexagonal array.

**Figure 3:**
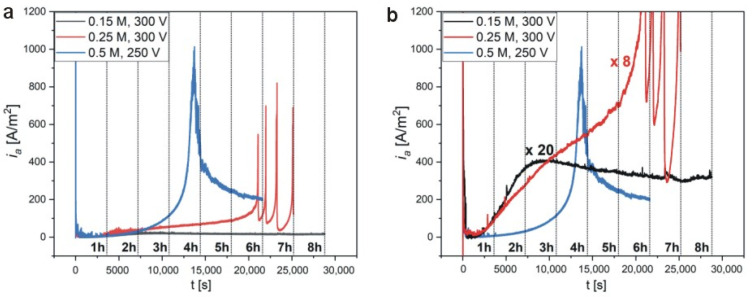
*i_a_*(*t*) curves recorded during anodization of aluminium at temperature of 5 °C in various malic acid concentrations and for various anodization times (**a**); the same *i_a_*(*t*) curves with amplified intensities for the samples anodized in 0.15 and 0.25 M malic solutions (**b**) (vertical lines indicate the subsequent hours of anodization).

The comparison of *i_a_*(*t*) curves (Figure 1) with corresponding SEM images of the resulted samples (Figure 2) allows one to speculate that the self-assembly of pores into a hexagonal pattern occurs after the appearance of the asymmetrical, broad peak in the *i_a_*(*t*) curve shown in Figure 1c. Since it can be expected that the appearance of this peak will be delayed with decreasing of electrolyte concentration, the anodizing time in 0.15 M and 0.25 M malic acid solutions was extended to 8 and 7, respectively. The process duration in 0.5 M solution was also extended from 5 to 6 h to observe the current evolution after the *i_a_* peak. The *i_a_*(*t*) curves for respective processes are shown in Figure 3. As can be observed, the curves are substantially different. Not only the intensity of the *i_a_* increases with malic acid concentrations, but the current flow has completely different character in all studied cases. Moreover, comparing the *i_a_* transients in Figure 1 and Figure 3 it can be noticed that, in contrast to the processes carried out in 0.25 M and 0.5 M, the anodization in 0.15 M electrolyte at 300 V is unrepeatable. Since the processes presented in Figure 3 were conducted in freshly prepared electrolytes, this observation indicates that the previous 0.15 M electrolyte has undergone serious changes due to the processes carried out at higher temperatures (25–10 °C), before the anodization at 5 °C. In the other two electrolytes, the changes are not observable, most probably due to a milder electrolyte modification, in which none (in 0.25 M) or only two (in 0.5 M) preceding processes were carried out before the last actual anodization. The problem with the electrolyte aging will be analyzed in more details in the next paragraphs.

Analyzing the *i_a_*(*t*) curves in Figure 3, it can be stated that the process conducted in 0.15 M is very similar to the ones observed during anodization in other organic electrolytes [19,20,21,33,34,35,36]. At the beginning of the process, the *i_a_* decreases rapidly to the lowest value (within few seconds) after passing through a current overshoot, and then it starts to grow to a maximum value (ca. 25 A/m^2^). After reaching the maximum, the *i_a_* begins to decrease very slowly to an equilibrium value. As noticed by Ma et al. [20,21], the *i_a_*(*t*) combines typical characteristics of mild (MA) and hard (HA) anodization, however, the pore nucleation during anodizing in organic electrolytes requires much longer time to initiate (minutes to hours as compared to seconds in a classical MA [8]). The pore evolution before and after the peak was investigated previously and was associated with various stages of pore nucleation process [20,21]. In the case of anodization in citric electrolyte, it was noticed that the amount of free citric acid anions play a crucial role in pore nucleation. The citric acid anions (i.e., H_2_Cit^−^, HCit^2−^, Cit^3−^) can chelate to Al^3+^ and form stable Al-citrate complexes. Ma et al. [21] have postulated that at the beginning of the process, a certain amount of Al^3+^ are consumed by the citrate anions to form the Al-citrate complexes, causing the *i_a_* to decrease. As the process proceeds, these complexes transform slowly to citric acid incorporated alumina, which randomly precipitate on barrier-type alumina to form protuberances. Depositing of the citric acid incorporated alumina on the whole alumina surface takes a long time. The high electric field concentrates between those protuberances, giving rise to field-assisted oxide dissolution accompanied by the *i_a_* increase, and finally to the pore development [21].

All the stages observed during anodization in the 0.15 M solution can also be distinguished in the process carried out in 0.5 M electrolyte, with the differences that the maximum in *i_a_* curve appears much later (after more than 3 h of anodization) and reaches much higher current value. After reaching the peak, the current decay occurs also much faster and it stabilizes at a greater *i_a_* value (Figure 3b). As stated previously, the commencement of the current growth to a maximal value indicates the beginning of pore formation process [21,24]. It was shown before that the pore nucleation in malic acid starts at grain boundaries of the aluminum [24]. During the *i_a_* increase up to the peak, the pores form islands separated by flat regions where no nano-dents are present. The gradual decrease of the *i_a_* after the peak is accompanied by the spread of pores over the entire surface of the aluminum substrate [24]. Prolonged anodization can lead to formation of the hexagonal close-packed pore arrays, but not necessarily. As discussed above, for the self-ordering regime to occur, high values of *i_a_* are required. The time of the peak occurrence, and thus the commencement of the pore formation, is different for various organic electrolytes. By comparison, the current peak appears after approximately 5–15 min of anodization in squaric acid [34], 25–40 min in tartaric acid [19] depending on applied voltage, 30–60 min in citric acid depending on electrolyte concentration [20], or 10–120 min in acetylenedicarboxylic acid depending on anodizing temperature [36]. The different times needed to form pores may be related with the dissociation constants (pK_a_) of the organic acids. The pK_a_ for squaric acid is 1.5, for tartaric—3.036, for citric acid—3.128, for acetylenedicarboxylic—1.75, and for malic acid—3.459 [22]. Hence, the malic acid represents the greatest pK_a_ values among those organics, which can delay the process of pore formation, because the smaller number of acid anions can be created under a given anodization condition. In addition, it is known that the amount of free acid anions is very important in pore nucleation process.

The anodization in 0.25 M is quite unusual. At the end of the 6th h of anodization, the current begins to jump, which is manifested in the appearance of four current spikes of relatively high intensity. The current behavior is most probably due to a sudden and temporary disruption of the barrier layer, which is almost immediately rebuilt, as reflected in a very fast *i_a_* drop to local minimums [37].

**Figure 4:**
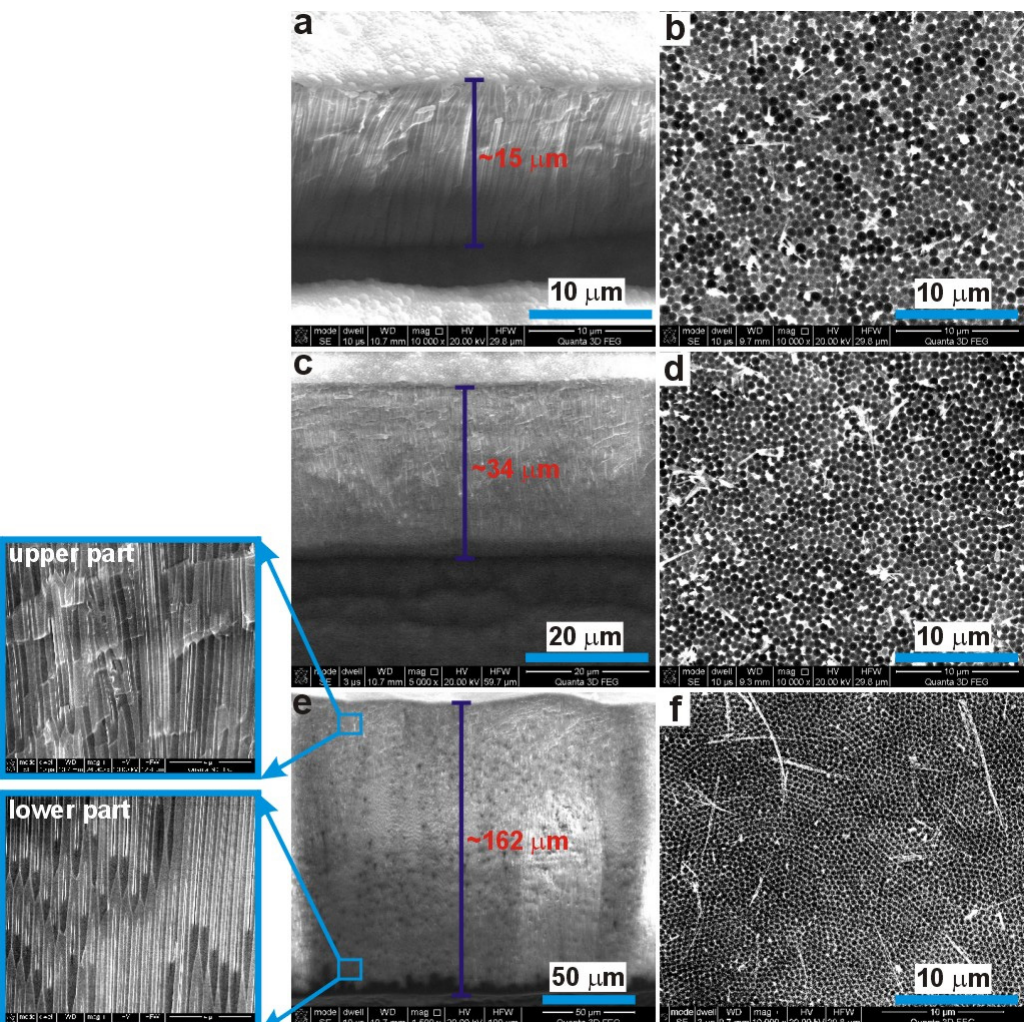
The SEM images of a cross-sectional view of AAO (**a**,**c**,**e**) and corresponding nanoconcaves left on aluminum surface after AAO dissolution (**b**,**d**,**f**) for the samples anodized under 300 V for 8 h in 0.15 M (**a**,**b**), 300 V for 7 h in 0.25 M (**c**,**d**), and 250 V for 6 h in 0.5 M (**e**,**f**) malic acid solutions at 5 °C.

The SEM images of cross-sectional views of AAO prepared during the anodization in 0.15 M, 0.25 M, and 0.5 M for 8, 7, and 6 h, respectively, and the corresponding nanoconcaves left on Al surface after the AAO dissolution, are shown in Figure 4. As could be expected, the thickness of AAO obtained in 0.25 M is about two times larger than the thickness of AAO produced in 0.15 M. However, the thickness of AAO membrane obtained in 0.5 M malic acid solution is one order of magnitude larger than the thickness of the other two AAO matrices, despite the fact that this membrane was produced under lower potential (250 V), for the shortest time (6 h), and all three processes were conducted at the same temperature (5 °C). This suggests that the concentration of malic acid is crucial for AAO growth and pore formation mechanism. In Figure 4b,d,f, the Al concaves are presented. As in the case of the 5-h anodization, the *D_c_* decreases with electrolyte concentration, which indicates the pore arrangement improvement and their gradual organization into close-packed hexagonal structure. Furthermore, the pores arrangement is still irregular in the samples anodized in lower acid concentration: The extension of anodization time did not help to organize the pores into the hexagonal close-packed arrays. Yet, the self-organized hexagonal ordering is maintained in the sample anodized in 0.5 M for 6 h. In addition, the closer inspection of the upper and lower part of the AAO membrane produced in 0.5 M solution (the SEM images in the blue squares in Figure 4e) revealed that the pores become much more parallel as the anodization proceeds (the lower part of AAO).

**Figure 5:**
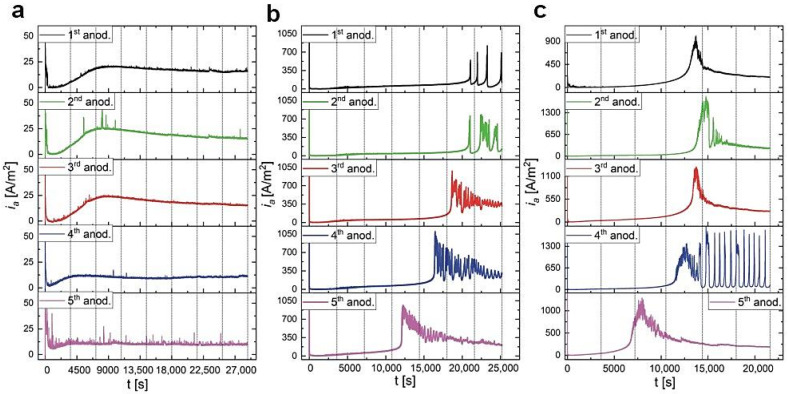
*i_a_*(*t*) curves recorded during anodization of aluminum in 0.15 M, 300 V (**a**), 0.25 M, 300 V (**b**), and 0.5 M, 250 V (**c**) malic acid concentrations and at temperature of 5 °C—the corresponding processes were repeated five times in a row in the same electrolyte (vertical dotted lines indicate the subsequent hours of anodization).

As previously suggested, the AAO growth during anodization in organic acids is associated with a consumption of organic anions. Regarding the example of anodization in citric acid, it was hypothesized that citrate anions can form complexes with Al^3+^ and these complexes transform slowly to citric acid incorporated alumina. Since malate anions can also form metal complexes [38,39], similar phenomena may occur during anodization in malic acid solutions. The anion consumption will certainly modify the electrolyte composition, which was indirectly confirmed by the initial experiments: The *i_a_*(*t*) curves in Figure 1 and Figure 3 are quite different, despite the same electrochemical conditions applied during those processes (0.15 M concentration, 300 V, 5 °C). Since organic anions participate in AAO formation, it can be thus assumed that decreasing the amount of the anions in the electrolyte will change the pore nucleation process. This, in turn, should be reflected in the course of *i_a_*(*t*) curves. To corroborate this assumption, the anodization processes in 0.15 M, 0.25 M, and 0.5 M solutions were repeated five times in a row in the same electrolyte. The resulted *i_a_*(*t*) curves are shown in Figure 5. As expected, the process is not repeatable when continued in the same electrolyte. In a 0.15 M solution, the changes are quite mild and are mainly manifested in a decrease of current intensity in the 4th and 5th anodization cycles (Figure 5a). In 0.25 M solution, the shape of *i_a_*(*t*) curves changes basically after each subsequent anodization cycle (Figure 5b). The onset of current growth shifts from the 5th to 6th h of anodization in the first process to the 3rd to 4th h of anodization in the fifth process (Figure 5b). Moreover, the sharp current spikes in the first process gradually transform into a turbulent current decay after reaching the first peak in *i_a_*(*t*). Then, also the current decay becomes less and less turbulent. As a result, the shape of the last *i_a_*(*t*) curve in the 0.25 M starts to resemble the *i_a_*(*t*) curves recorded during anodization in 0.5 M solution. In a 0.5 M malic acid solution, a similar trend of the current behavior is observed, although the onset of current growth begins to shift to the left only from the 4th anodization cycle: The *i_a_* maximum is located between the 3rd and 4th h of anodization in the first cycle and between the 2nd and 3rd h of anodization in the fifth cycle (Figure 5c). Moreover, in the 4th cycle, during the current decay, sharp but fairly regular current spikes occur, similar to these observed during anodization on 0.25 M in the 1st cycle. The origin of these spikes is not entirely known but, as mentioned already, it can result from a local, temporary breakdown and immediate reconstruction of barrier layer. In general, it can be stated that, most probably owing to a drastic consumption of anions participating in the alumina formation, the anodization process in malic acid solution is not reproducible when conducted in the same electrolyte. Furthermore, the aging of malic acid electrolyte proceeds very fast: actually the changes in electrolyte are visible already in the second anodizing round.

The current evolution with anodization cycles was not investigated before and can be tentatively explained by invoking the fact that *i_a_* is actually a combination of two types of current density: ionic current density (*i_i_*) and electronic current density (*i_e_*) [40]. The *i_i_* participates directly in AAO formation, and *i_e_* influences directly the stability of the anodization process. To the *i_i_* contribute inward migrating O^2−^ and acid anions, such as malate anions in the present case. Under high voltage anodization in malic acid, the malate anions are attracted by the anode (Al). It is, therefore, possible that in high-concentrated electrolytes (0.25 M and 0.5 M), the anion migration is hindered by a high amount of Al(III)-malate complexes formed under the high voltage. Owing to a screening effect of the Al(III)-malate complexes (and/or malate anions), the current growth and thus the pore formation is delayed. When the amount of anions is decreasing due to their incorporation into the AAO framework, the screening effect becomes less effective, and the current growth and pore formation starts earlier. In lower malic acid concentration (0.15 M), the amount of anions does not screen the anion attraction by the anode and the overall current simply decreases with anodization cycles, owing to a progressive consumption of the anions by the growing AAO.

**Figure 6:**
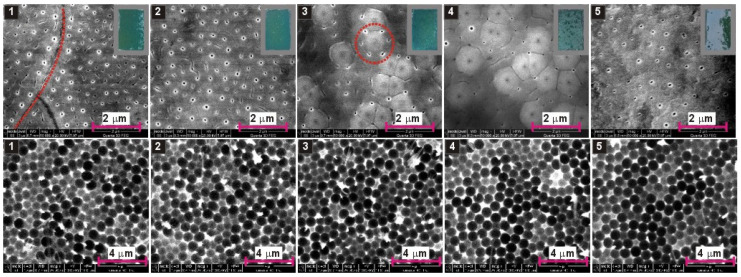
SEM images of AAO—top view (**upper row**) and nanoconcaves left on aluminum surface after AAO dissolution (**lower row**) for the samples anodized for 8 h, at 5 °C, 300 V, five times in a row in the same 0.15 M malic acid solution; in the right corners of the SEM images presented in the upper row, photographs of the samples after anodization are shown (in grey frames).

**Figure 7:**
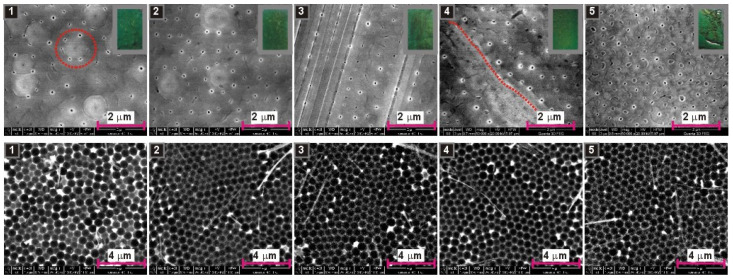
SEM images of AAO—top view (**upper row**) and nanoconcaves left on aluminum surface after AAO dissolution (**lower row**) for the samples anodized for 7 h, at 5 °C, 300 V, five times in a row in the same 0.25 M malic acid solution; in the right corners of the SEM images presented in the upper row, photographs of the samples after anodization are shown (in grey frames).

**Figure 8:**
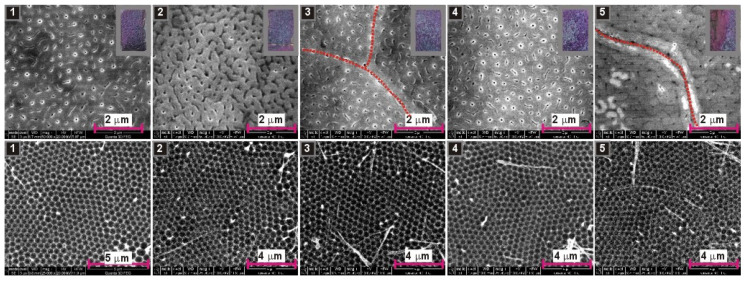
SEM images of AAO—top view (**upper row**) and nanoconcaves left on aluminum surface after AAO dissolution (**lower row**) for the samples anodized for 6 h, at 5 °C, 250 V, five times in a row in the same 0.5 M malic acid solution; in the right corners of the SEM images presented in the upper row, photographs of the samples after anodization are shown (in grey frames).

In Figure 6, Figure 7 and Figure 8, SEM images of resulted AAO and Al surface after the AAO dissolution prepared in various malic acid solutions in the subsequent five anodization cycles, are shown. In addition, the photographs of AAO membranes are demonstrated in gray frames (in the upper, right corner of the AAO SEM images). As can be seen, the oxides prepared in 0.15 M and 0.25 M solutions have blue-green color, which indicate the incorporation of the entities that absorb light in the red regions of the visible light spectrum. The oxides prepared in 0.5 M instead have violet color, which suggest the presence of the entities that absorb light from green to yellow regions of the visible light spectrum. However, the change of colour can also be related with much larger AAO thickness in the samples synthesized in the electrolyte of 0.5 M concentration (Figure 4). In addition, the AAO layers are generally mechanically stable (do not peel-off from Al substrates). The visible different shades of green and violet colours on the photographs indicate probably an inhomogeneous thickness of the AAO layers on the anodized surface. During anodization in citric acid, black AAO was formed [20,21]. The black oxide was related with incorporation of carbon-bearing elements, such as carbonates, carboxylates, carbonyls of aluminum, or amorphous carbon into the AAO walls [21,41,42]. The violet–blue–green colouration of AAO fabricated in malic acid may indicate the incorporation of Al(III)–malic acid complexes into the AAO framework, since some metal–malate complexes demonstrate the absorption in the visible part of the spectrum [43,44].

Top surface of AAO bears rather common features in all analyzed samples. Small pores are, in general, randomly distributed; however, they tend to organize along grain borders (red dotted lines in selected SEM images), which confirms the previous observations that the pore nucleation initiates at grain boundaries of the aluminum [24]. Furthermore, some clusters of nanopores surround distinct globular and bright spots visible on AAO surface (highlighted by red dotted circles in selected SEM images), suggesting that there are other sites that promote pore nucleation. These spots may be associated with the protuberances caused by agglomerated Al-malate complexes incorporated into alumina nanoparticles, which precipitate on the formed alumina surface. This mechanism of pore growth was proposed in the citric acid electrolyte [15], but can be also true in this case.

Analyzing nano-concaves on Al surface, being a replica of AAO pore bottom, it can be seen that there is tiny progress in pore organization with the number of cycles in the samples anodized in 0.15 M solution (Figure 6). In the sample anodized in 0.5 M, the arrangement of pores does not improve visibly with the number of cycles, because the pores were well organized already in the first cycle and (Figure 8). Furthermore, the sample anodized in the 4th cycle, where the sharp and regular current spikes were observed (Figure 5c), does not reflect any clear signs of the unusual current behavior: The nano-concaves are as regular as in the other samples prepared in 0.5 M solution. However, in the samples anodized in 0.25 M solution, the improvement of pore arrangement is evident: The pores start to organize into hexagonal close-packed structure already in the second anodization cycle (Figure 7). To the best of our knowledge, this is the first observation of this kind: mere repetition of the process in the same electrolyte generates such internal changes in this electrolyte that induce self-ordering of pores. The evolution of pore arrangement can be associated with the *i_a_*(*t*) behavior in Figure 5b: The *i_a_*(*t*) curves start to develop a shape similar to that recorded for the sample anodized in 0.5 M solution, confirming thus that the broad, unsymmetrical but intensive peak in *i_a_*(*t*) curve is an indicator of the appearance of a self-ordering regime during anodization in malic acid electrolytes. Although more research is needed to clarify this issue, at this point it can be stated that anodization in malic acid is challenging in terms of process controlling by sole external electrochemical parameters (applied voltage, temperature, etc.). Anodization reproduced under exactly the same electrochemical conditions can result in different current evolution and pore formation.

**Figure 9:**
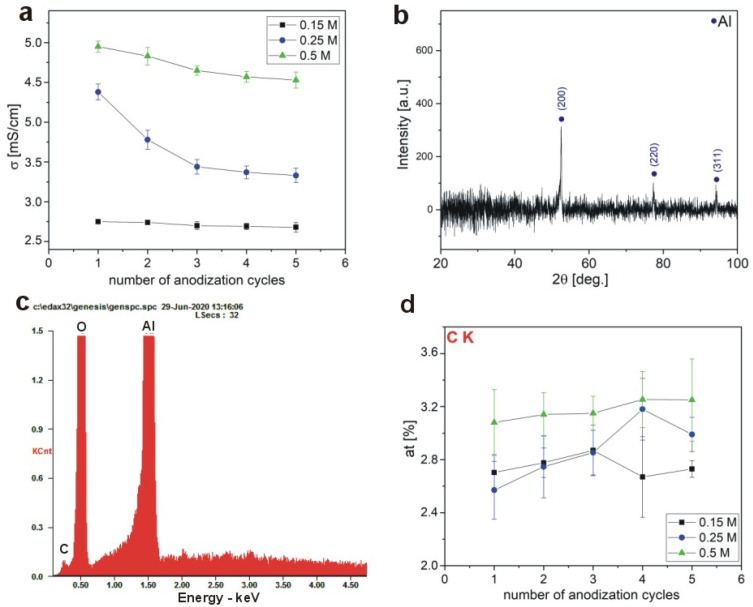
Electric conductivity (σ) as a function of anodization cycles (**a**); XRD pattern of AAO prepared during anodization in the 0.5 M malic acid solutions at 5 °C for 6 h (**b**); an exemplary EDS spectrum (**c**); and EDS carbon analysis of AAO as a function of anodization cycles (**d**) for AAO obtained during anodization for 8, 7, and 6 h in the 0.15 M, 0.25 M, 0.5 M malic acid solutions, respectively, at 5 °C.

A consumption of organic anions, which are the source of the carbon-bearing elements, should be reflected in conductivity (σ) of electrolyte [mS·cm^−1^]. In Figure 9a, the σ, as a function of the number of anodization cycles for each electrolyte concentration, is demonstrated. The curves in Figure 9a show a typical relationship between conductivity and concentration: The σ increases as the electrolyte concentration increases. Yet, it can be seen that for each malic acid concentration, the σ decreases with the number of cycles, implying decreasing amounts of anionic species in a given electrolyte. Therefore, the data indirectly support the assumption that the malate anions are embodying in the alumina during the anodization in malic acid. In Figure 9b–d, an exemplary XRD pattern, EDS spectrum and the EDS carbon (C) analysis of respective AAOs, respectively, are shown. The formed AAOs are amorphous as observed based on the XRD analysis of the sample prepared in the highest malic acid solution. In the XRD pattern, only the peaks from the Al substrate (the reflection from the (200), (220), and (311)) are present (PDF Card 01-071-3760). The at.% of C in the AAO prepared in 0.15 M and 0.25 M is comparable, but it increases slightly in the AAO fabricated in 0.5 M solution. However, the amount of C does not show any obvious dependence on the number of cycles in the AAO prepared in different malic acid concentrations, implying that the incorporation of the C-bearing elements into the AAO framework is rather constant in the succeeding anodization process. This is not surprising though since the driving force (the applied potential) that attracts the anions to Al, as well as anodizing temperature, were constant in every cycle for a given malic acid concentration.

**Figure 10:**
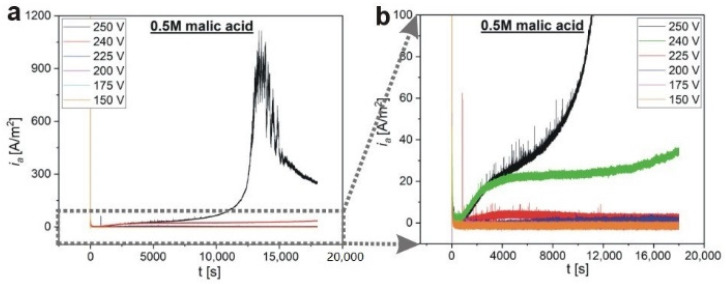
The *i_a_*(*t*) curves for anodization in 0.5 M malic acid solution for 5 h, at 5 °C, and under various applied voltages (150–250 V) (**a**); a larger magnification of the *i_a_*(*t*) curves (**b**).

The influence of the applied voltage on pore formation during anodization in 0.5 M malic acid solution, at 5 °C, was also studied. In Figure 10, the *i_a_*(*t*) curves recorded at voltages 150–250 V are presented. The striking feature of those curves is a huge difference between the *i_a_* when the voltage decreases from 250 V to already 240 V. In the latter case, the *i_a_* does not reach the value of 40 A/m^2^ within the whole 5-h process, while the peak in the *i_a_*(*t*) curve recorded during anodization at 250 V reached ≈1000 A/m^2^. Anodization under constant voltage < 240 V does not generate large current values, and at 150 V the *i_a_* oscillates around 0.5–1 A/m^2^. The huge drop in the current density may be ascribed to the incorporation of malate containing species into alumina, which causes the electrolyte impoverishment (aging) out of acid anions as discussed above. It can also indicate that anion migration to the anode requires sufficiently high electric field strength (anodizing potential) to contribute to *i_i_*, and thus to *i_a_*. It seems thus that the voltage range of 240–250 V defines a suitable processing window to form regular pores during anodization in 0.5 M malic acid solution at 5 °C.

**Figure 11:**
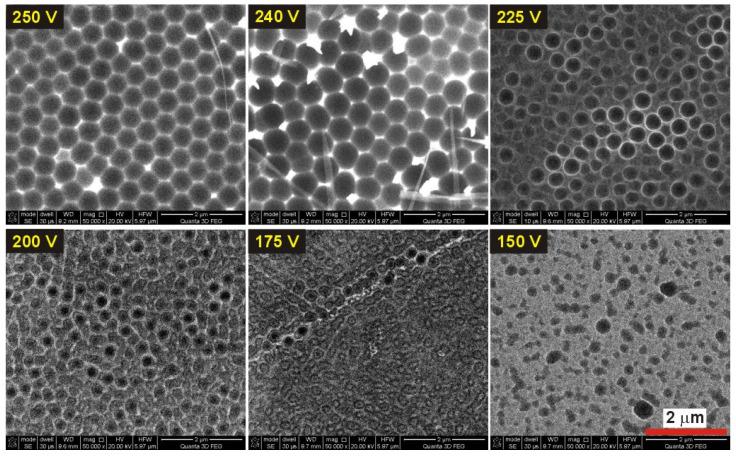
SEM images (magnification 50,000×) of the nanoconcave arrays obtained after removal of AAO in the samples anodized under various applied voltage in 0.5 M malic acid solution for 5 h, at 5 °C (the red scale bar = 2 μm in the bottom right corner is for all images).

In Figure 11, SEM images of the corresponding Al surface obtained after dissolution of alumina, are presented. It can be noticed that up to 240 V, the Al surface lacks a signature of typical imprint coming from porous AAO. After a 5-h anodization at 225 V, pores become more circular and start to develop in close proximity to each other, but are still disordered. Only at 240 V a typical replica of the bottom part of AAO is formed on Al surface. The hexagonal pore arrangement is, however, worse in this sample as compared to that in the sample anodized under 250 V. Owing to weaker self-organization conditions, the distance between the centers of nanoconcaves (*D_c_*) is also larger: 586 ± 45 nm as compared to 527 ± 12 nm in the sample anodized at 250 V, where close-packed hexagonal pore arrays are clearly visible.

## 4. Conclusions

In this work, Al foils were anodized in malic acid electrolytes of different concentrations: 0.15 M, 0.25 M, and 0.5 M. It was shown that, in agreement with the previous findings, the lower the temperature and the higher the concentration of the electrolyte, the closer are the conditions to the self-ordering regime. The close-packed hexagonal pore structure was formed in 0.5 M solution, at 250 V and temperature of 5 °C. Moreover, the anodization was investigated as a function of the number of cycles performed in the same electrolyte. It was demonstrated that both current density, *i_a_*(*t*), and pore formation process are different in each and subsequent anodization cycle. The differences were most pronounced in the higher concentrated malic acid solutions. First of all, the onset of the *i_a_*(*t*) growth to a maximum begins to appear earlier on the next anodization cycle carried out in the same electrolyte. This phenomenon was tentatively explained by fast electrolyte aging after subsequent anodization, which is most probably caused by a massive incorporation of malic acid complexes into the AAO framework. The impoverishment of the electrolyte out of the malate anions changes internal electrolyte conditions, making easier the attraction of the malate anions to the Al anode. Moreover, in a 0.25 M solution, strong current spikes at the end of the 7-h process in the first cycle transforms into one unsymmetrical, broad and intensive peak in the fifth cycle, and the whole *i_a_*(*t*) curve starts to resemble the curve recorded during anodization in 0.5 M. As an effect, already after the second anodization cycle, the pores start to form regular, hexagonal close-packed arrays. At the end, the influence of applied voltage on AAO growth was analyzed in 0.5 M solution. It was shown that at a temperature of 5 °C, the voltage range of 240–250 V is a suitable processing window to produce AAO with typical hexagonal pore arrangement. Below 240 V, no characteristic imprint coming from porous AAO was developed.

## References

[1] Nie X.L., Wang L., Northwood D.O. (2005). Corrosion protection of anodic oxide coatings on an Al-Si alloy. Surf. Coat. Technol..

[2] Whelan M., Tobin E., Cassidy J., Duffy B. (2016). Optimisation of anodic oxidation of aluminium for enhanced adhesion and corrosion properties of sol-gel coatings. J. Electrochem. Soc..

[3] Bouchama L., Azzouz N., Boukmouche N., Chopart J.P., Daltin A.L., Bouznit Y. (2013). Enhancing aluminum corrosion resistance by two-step anodizing process. Surf. Coat. Technol..

[4] Md Jani A.M., Losic D., Voelcker N.H. (2013). Nanoporous anodic aluminium oxide: Advances in surface engineering and emerging applications. Prog. Mater. Sci..

[5] Santos A., Kumeria T., Losic D. (2013). Nanporous anodic aluminum oxide for chemical sensing and biosensors. TrAC Trends Anal. Chem..

[6] Lee W., Park S.-J. (2014). Porous anodic aluminum oxide: Anodization and templated synthesis of functional nanostructures. Chem. Rev..

[7] Larosa C., Terencio T., Converti A., Eggenhöffer R. (2014). Anodic porous alumina array for cyanine fluorophore Cy3 confinement. J. Mater. Sci. Nanotechnol..

[8] Sulka G.D., Eftekhari A. (2008). Highly ordered anodic porous alumina formation by self-organized anodizing. Nanostructures Materials in Electrochemistry.

[9] Stępniowski W.J., Bojar Z., Aliofkhazraei M., Makhlouf A.S.H. (2016). Nanoporous anodic aluminium oxide: Fabrication, characterization, and applications. Handbook of Nanoelectrochemistry: Electrochemical Synthesis Methods, Properties, and Characterization Techniques Part II.

[10] Jagminas A., Vrublevsky I., Sulka G.D. (2020). Chapter three—Anodizing of aluminum under the burning conditions. Nanostructured Anodic Metal Oxides.

[11] Su Z., Zhou W., Jianga F., Hong M. (2012). Anodic formation of nanoporous and nanotubular metal oxides. J. Mater. Chem..

[12] Jessensky O., Müller F., Gösele U. (1998). Self-organized formation of hexagonal pore arrays in anodic alumina. Appl. Phys. Lett..

[13] Nielsch K., Choi J., Schwirn K., Wehrspohn R.B., Gösele U. (2002). Self-ordering regimes of porous alumina: The 10% porosity rule. Nano Lett..

[14] Kikuchi T., Nishinaga O., Natsui S., Suzuki R.O. (2015). Fabrication of self-ordered porous alumina via etidronic acid anodizing and structural color generation from submicrometer-scale dimple array. Electrochim. Acta.

[15] Takenaga A., Kikuchi T., Natsui S., Suzuki R.O. (2016). Exploration for the self-ordering of porous alumina fabricated via anodizing in etidronic acid. Electrochim. Acta.

[16] Lee W., Ji R., Gösele U., Nielsch K. (2006). Fast fabrication of long-range ordered porous alumina membranes by hard anodization. Nature.

[17] Norek M., Dopierała M., Bojar Z. (2016). The influence of pre-anodization voltage on pore arrangement in anodic alumina produced by hard anodization. Mater. Lett..

[18] Kikuchi T., Nakajima D., Nishinaga O., Natsui S., Suzuki R.O. (2015). Porous aluminum oxide formed by anodizing in various electrolyte species. Curr. Nanosci..

[19] Ono S., Saito M., Asoh H. (2005). Self-ordering of porous alumina formed in organic acid electrolytes. Electrochim. Acta.

[20] Ma Y., Wen Y., Li J., Li Y., Zhang Z., Feng C., Sun R. (2016). Fabrication of self-ordered alumina films with large interpore distance by Janus anodization in citric acid. Sci. Rep..

[21] Ma Y., Wen Y., Li J., Lu J., Li Y., Yang Y., Feng C., Hao C., Zhang Z., Hu J. (2018). Pore nucleation mechanism of self-ordered alumina with large period in stable anodization in citric acid. J. Electrochem. Soc..

[22] Martell A.E., Smith R.M. (1976). Critical Stability Constants.

[23] Qin X., Zhang J., Meng X., Deng C., Zhang L., Ding G., Zeng H., Xu X. (2015). Preparation and analysis of anodic aluminum oxide films with continuously tunable interpore distances. Appl. Surf. Sci..

[24] Kikuchi T., Yamamoto T., Suzuki R.O. (2013). Growth behavior of anodic porous alumina formed in malic acid solution. Appl. Surf. Sci..

[25] Chu S.Z., Wada K., Inoue S., Isogai M., Katsuta Y., Yasumori A. (2006). Large-scale fabrication of ordered nonporous alumina films with arbitrary pore intervals by critical-potential anodization. J. Electrochem. Soc..

[26] Horcas I., Fernandez R., Gomez-Rodriguez J.M., Colchero J., Gomez-Herrero J., Baro A.M. (2007). WSXM: A software for scanning probe microscopy and a tool for nanotechnology. Rev. Sci. Instrum..

[27] Lee W., Nielsch K., Gösele U. (2007). Self-ordering behavior on nanoporous anodic aluminum oxide (AAO) in malonic acid anodization. Nanotechnology.

[28] Shingubara S., Okino O., Sayama Y., Sakaue H., Takahagi T. (1997). Ordered two-dimensional nanowire array formation using self-organized nanoholes of anodically oxidized aluminum. Jpn. J. Appl. Phys..

[29] Sul Y.-T., Johansson C.B., Jeong Y., Albrektsson T. (2001). The electrochemical oxide growth behavior on titanium in acid and alkaline electrolytes. Med. Eng. Phys..

[30] Li Y., Zheng M., Ma L., Shen W. (2006). Fabrication of highly ordered nanoporous alumina films by stable high-field anodization. Nanotechnology.

[31] Ono S., Saito M., Ishiguro M., Asoh H. (2004). Controlling factor of self-ordering of anodic porous alumina. J. Electrochem. Soc..

[32] Ono S., Saito M., Asoh H. (2004). Self-ordering of anodic porous alumina induced by local current concentration: Burning. Electrochem. Solid State Lett..

[33] Stojadinovic S., Belca I., Tadic M., Kasalica B., Nedic Z., Zekovic L.j. (2008). Galvanoluminescence properties of porous oxide films formed by anodization of aluminum in malonic acid. J. Electroanal. Chem..

[34] Kikuchi T., Yamamoto T., Natsui S., Suzuki R.O. (2014). Fabrication of anodic porous alumina by squaric acid anodizing. Electrochim. Acta.

[35] Takenaga A., Kikuchi T., Natsui S., Suzuki R.O. (2015). Self-ordered aluminum anodizing in phosphonoacetic acid and its structural coloration. ECS Solid State Lett..

[36] Kikuchi T., Nishinaga O., Natsiu S., Suzuki R.O. (2014). Fabrication of anodic nanoporous alumina via acetylenedicarboxylic acid anodizing. ECS Electrochem. Lett..

[37] Elabar D., La Monica G.R., Santamaria M., Di Quarto F., Skeldon P., Thompson G.E. (2017). Anodizing of aluminium and AA 2024-T3 alloy in chromic acid: Effects of sulphate on film growth. Surf. Coat. Technol..

[38] Panina N.S., Davydova M.K., Nikandrov E.M., Ruzanov D.O., Belyaev A.N. (2019). Formation of metal complexes with malate anions: Quantum-chemical modeling. Russ. J. Inorg. Chem..

[39] Venturini-Soriano M., Berthon G. (2001). Aluminum speciation studies in biological fluids Part 7. A quantitative investigation of aluminium(III)—Malate complex equilibria and their potential implications for aluminium metabolism and toxicity. J. Inorg. Biochem..

[40] Yi L., Zhiyuan L., Xing H., Yisen L., Yi C. (2001). Formation and microstructures of unique nanoporous AAO films fabricated by high voltage anodization. J. Mater. Chem..

[41] Jagminas A., Kaciulis S., Klimas V., Reza A., Mickevicius S., Soltani P. (2016). Fabrication of graphene-alumina heterostructures films with nanotube morphology. J. Phys. Chem. C.

[42] Chernyakova K., Karpicz R., Zavadski S., Poklonskaya O., Jagminas A., Vrublesky I. (2017). Structural and fluorescence characterization of anodic alumina/carbon composite formed in tartaric acid solution. J. Lumin..

[43] SlideServe. https://www.slideserve.com/tiva/common-valence-states-of-chromium.

[44] Taube F., Drobot B., Rossberg A., Foestendorf H., Acker M., Patzschke M., Trumm M., Taut S., Stumpf T. (2019). Thermodynamics and structural studies on the Ln(III)/An(III) malate complexation. Inorg. Chem..

